# Ethics of Vaccination in Childhood—A Framework Based on the Four Principles of Biomedical Ethics

**DOI:** 10.3390/vaccines9020113

**Published:** 2021-02-02

**Authors:** Meta Rus, Urh Groselj

**Affiliations:** 1 University Children’s Hospital, University Medical Centre Ljubljana, Bohoričeva 20, 1000 Ljubljana, Slovenia; meta.rus9@gmail.com; 2 Department of Endocrinology, Diabetes and Metabolic Diseases, University Children’s Hospital, University Medical Centre Ljubljana, Bohoričeva 20, 1000 Ljubljana, Slovenia; 3 Faculty of Medicine, University of Ljubljana, Vrazov trg 2, 1000 Ljubljana, Slovenia; 4 National Medical Ethics Committee of Republic of Slovenia, Ministry of Health, Štefanova 5, 1000 Ljubljana, Slovenia

**Keywords:** vaccination, children, bioethics, principles of biomedical ethics, autonomy, nonmaleficence, beneficence, justice, common good, herd immunity

## Abstract

Although vaccination is recognised as the top public health achievement of the twentieth century, unequivocal consensus about its beneficence does not exist among the general population. In countries with well-established immunisation programmes, vaccines are “victims of their own success”, because low incidences of diseases now prevented with vaccines diminished the experience of their historical burdens. Increasing number of vaccine-hesitant people in recent years threatens, or even effectively disables, herd immunity levels of the population and results in outbreaks of previously already controlled diseases. We aimed to apply a framework for ethical analysis of vaccination in childhood based on the four principles of biomedical ethics (respect for autonomy, nonmaleficence, beneficence and justice) to provide a comprehensive and applicable model on how to address the ethical aspects of vaccination at both individual and societal levels. We suggest finding an “ethical equilibrium”, which means that the degree of respect for parents’ autonomy is not constant, but variable; it shall depend on the level of established herd immunity and it is specific for every society. When the moral obligation of individuals to contribute to herd immunity is not fulfilled, mandatory vaccination policies are ethically justified, because states bear responsibility to protect herd immunity as a common good.

## 1. Introduction

Although vaccination is recognised as the top public health achievement of the twentieth century, saving millions of individual lives and, importantly, prolonging life expectancy [1], the general consensus about its beneficence has not been reached among people [2,3,4]. In countries with well-established immunisation programmes, vaccines are said to be “victims of their own success”, because low incidences of diseases now prevented with vaccines has diminished the experience of historical burdens of several devastating communicable diseases [2]. Nowadays, a wide variety of opinions about vaccination exist; some people are against it in principle, others are against its mandatoriness or against the involvement of the state; yet others are just concerned about its safety issues and maybe prefer alternative vaccination programmes or delayed vaccination [5,6,7]. Therefore, different terms for parents who lack compliance with vaccination are used, though not consistently [6,8,9,10]. The term “anti-vaxxers” refers to a broad group of people, who are against vaccination for whatever reason [6]. Terms “vaccine-refusal” or “vaccine-reluctancy” represent the anti-vaccination extreme and define individuals who fail to vaccinate themselves or their children for different reasons [6,11]. “Vaccine-hesitancy” is a term that covers the continuum of opinions between pro- and anti-vaccination extremes. Vaccine-hesitant individuals do not refuse vaccination in principle but are concerned about its safety/efficiency or maybe just prefer alternative vaccination schedules [2,6,12]. In our review, we use the term “vaccine-hesitant parents”, because it applies to the whole spectrum of different opinions about vaccination.

The number of vaccine-hesitant people is high, with up to 90% of people expressing hesitancy in at least one aspect of vaccination, using the World Health Organisation Strategic Advisory Group of Experts (WHO SAGE) Vaccine Hesitancy Scale [13,14,15]. However, a large-scale retrospective study, published in 2020 in The Lancet, reported that confidence in the importance, safety and effectiveness of vaccines improved in some countries across the world and fell in the others in the last few years [16]. The percentage of vaccine-refusing people is lower than vaccine-hesitant and is estimated to around 5% [17]. Vaccine hesitancy is, especially in high-income countries, one of the most important reasons for lower immunisation coverage, which results in outbreaks of vaccine-controllable diseases [2,7,18,19]. Due to high contingency, several measles outbreaks across Europe and North America in recent years raised attention to low vaccination coverage in particular countries [15,20,21]. WHO declared vaccine hesitancy, defined as the reluctance or refusal to vaccine despite the availability of vaccines, as one of the world’s top ten global health threats in 2019 [22]. There are many reasons why parents might be hesitant or refuse to vaccinate their children, including worries about a vaccine’s safety and the maturity of a child’s immune system, absence of the disease in a certain population, motives of pharmaceutical industry, diminished trust in public health system, misinformation, perception of coercion, religious beliefs, etc. [2,7].

Preventive vaccination is a complex topic, because it involves an individual, doctor–patient perspective, as well as a public health perspective. At the level of society, the major ethical dilemmas concern vaccine development and safety issues [23,24,25], fair global vaccine allocation and development [26], vaccination for travellers and workers [21,27,28] and ethical justification of measures to maintain herd immunity of the population [5,15,19]. At the level of an individual, there can be conflicts between respect for parents’ autonomy, the child’s best interest and just contribution to herd immunity [5,29]. Although every country has its own vaccination policy and recommendations, each situation when treating children of vaccine-hesitant parents is unique and needs an individual and personal approach [30,31].

In this article, we develop a systematic approach by the four principles of biomedical ethics (the respect for individual’s autonomy, the principles of non-maleficence, beneficence and justice), first introduced in 1979 by Childress and Beauchamp, to be applicable as the ethical framework to facilitate the decision-making process. After the first release, the principles-based approach quickly became a dominant framework for American and also global bioethics [32,33]. However, there are many possible ways of approaching bioethical problems and none of them is ideal. Despite some criticism regarding the four-principles approach [34,35,36,37], the framework is widely accepted and compatible with a variety of moral theories [38]. Although the approach is basically simple, as ethics should be in order to serve different profiles of people, it can also be complexified [38]. Beauchamp and Childress claim that their framework captures major moral considerations that are essential starting points for biomedical ethics, but only the process of specification and balancing broad principles and rules leads to the concrete moral judgments [39]. The four principles theory, as also other bioethical theories, should maybe not be understood as action-guiding but rather as procedures by which one’s decisions can achieve a reasonable degree of moral justification [40]. The approach to ethics of vaccination, developed in this paper, could; therefore, help healthcare providers and also decision-makers at various levels to better understand ethical issues and values at stake, and suggest them a more systematic and better defined way of ethical deliberation to reach best possible solutions [31,41].

Thus, we aimed to apply a framework for ethical analysis of vaccination in childhood basing on the four principles of biomedical ethics to provide a comprehensive and applicable model on how to address the ethical aspects of vaccination at both individual and societal levels. The paper is primarily meant not to innovate or deepen the theory behind the ethics of vaccination, but to comprehensively explore the ethical dilemmas regarding childhood vaccination. This might help especially the healthcare workers, which daily encounter dilemmas regarding vaccination in their practices, to better understand the subject and get support for their work.

## 2. Respect for Autonomy

The principle of respect for autonomy has a variety of interpretations. In clinical ethics it is usually understood as a right of an individual patient or research subject to decide about themselves according to their own principles, which gives them also responsibilities for the possible outcomes [41,42,43,44]. This principle is a fundamental ethical and political concept especially in countries with western tradition over the last 50 years [41,42,43]. However, it is not an absolute principle and should not represent present-day individualism, as some critics have argued, but should always be balanced by other principles, taking into account social responsibilities and communal goals [39]. In practice, autonomy is exercised through informed consent or informed refusal [41,42]. There are at least four mandatory criteria that need to be fulfilled for its legitimacy: decision making capacity of a patient, adequate disclosure of information with its adequate understanding and voluntariness [41,45,46]. In the paediatric setting, when patients do not have appropriate decisional capacity for informed consent, two other important concepts were endorsed by the policy statement of the American Academy of Pediatrics (published in 1995, reaffirmed in 2016): parental permission and child’s assent [47,48]. The latter gains its value with child’s maturation, until full decision making capacity is assigned to an adolescent or young adult [47,49]. In the ethics of vaccination, the degree of respect for parents’ autonomy is one of the key issues at stake. In many cases, vaccine hesitancy or refusal is a consequence of insufficient or inappropriate information, its misunderstanding or presence of manipulation, which constrain one’s autonomy [2,31]. In this section, we will go through the main criteria for autonomous decision making from the perspective of vaccination.

### 2.1. Decision Making Capacity

**Figure 1:**
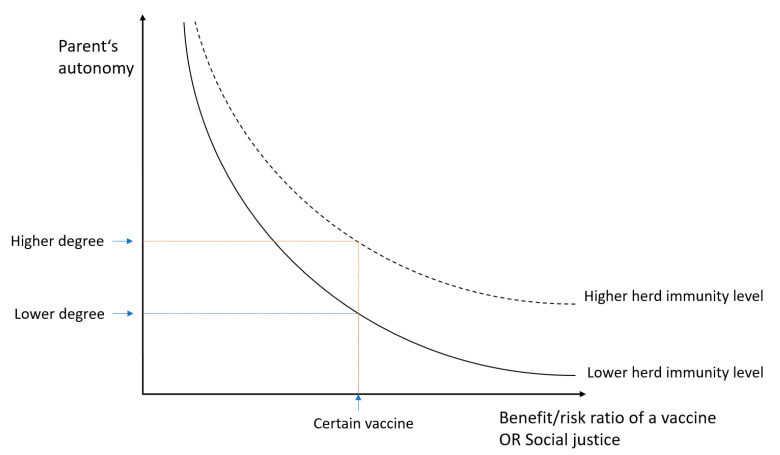
Relation between parents’ autonomy and benefit:risk ratio of a vaccine (or social justice). The curve can be moved upwards in case of a good society immunisation level, which could allow a higher degree of parents’ autonomy and non-medical exemptions from vaccination.

To make an informed consent or refusal, individuals need a minimum level of capacity to receive information, understand it, make their choices and articulate them. However, each person is owed disclosure of information according to their own health literacy, which is further discussed in the next paragraph. In contrast to competency, which is determined by court, capacity is task specific—depends on a context [41]. Regarding vaccination, it is mostly parents who make decisions on behalf of their children, because the majority of vaccinations are scheduled in early childhood [41,50]. However, because a state also has a duty and interest in protecting a child from harm, it can challenge parental authority in situations in which a child is put at risk (the doctrine of parens patriae) [47]. The degree of parent’s autonomy depends on a type of planned intervention (or a type of vaccine); the higher a ratio between benefit and burden (having in mind the principles of beneficence and non-maleficence), the less decisive parents’ autonomy is, and vice versa (Figure 1). The role of parental autonomy is also affected by the principle of justice (in the context of vaccination, the need to contribute to the herd immunity in a population is its important part, which will be dealt with in the Discussion section).

### 2.2. Disclosure of Information and Adequate Understanding

For parental permission, adequate explanation which parents or their surrogates understand needs to be provided [47]. Lack of information, misunderstanding or false information are common causes for vaccine hesitancy or refusal [2,4]. Studies have shown that parents who delayed or refused vaccines do not consider the child’s health care provider as a reliable information source and are more likely to seek vaccine information on the Internet [2,30,51]. Prior to burdening parents with responsibility, which comes together with autonomy, it is the responsibility of accountable public health structures and its professionals and also of individual clinicians to provide adequate, reliable and understandable information about vaccination [52]. Improved parents’ education and their adequate informing play a key role in the promotion of vaccination and enable the parents to make responsible immunisation decisions [53,54,55]. Trust of the society in scientific evidence about vaccines, in the health care system and in vaccination policy is crucial, but not self-evident. It needs to be restored and maintained by provision of transparent information about vaccines, consistent attitudes of scientists and health care professionals and by the government taking responsibility for individuals affected by side effects [29,56]. Confidence is both a means and a result of ethically justified and efficient public health activities [56]. Additionally, a trustful relationship between medical personnel and parents is also important. Listening carefully to parent’s concerns and provision of clear information about risks and benefits of vaccination can help hesitant parents to understand principles of vaccination and, with their consent, to obviate ethical dilemmas of vaccine refusal [31,56]. However, because time meant for consultation with parents prior to vaccination is limited and it is often not possible to address all the parents’ concerns, a systematic approach is preferable (e.g., by social media or in organized forms of education about vaccination) [57].

### 2.3. Voluntariness

A voluntary decision and absence of manipulation or coercion are an essential part of informed consent, though many factors may influence patient’s position (e.g., previous experience, social myths, opinion of family members and friends, clinician’s persuasion, legal obligations and constitutional pressures) [41,47]. Many of them do not threaten autonomous choice, even more, they are a fundamental part of decision making (so called “noncontrolling influence”) [41]. But some factors may limit one’s autonomy (“controlling influence”), and they also appear among reasons for vaccination hesitancy (i.e., conspiracy theories and family members’ pressure) [2,41]. These reasons need to be recognised and, if possible, avoided. A question arises, what is the relationship between autonomy and authority of behaviour prescribing organizations and traditions (i.e., government, religion, medical authority). Some claim that they are incompatible, but others see a conflict only when authority has not been properly delegated or accepted [41,58].

### 2.4. Conscientious Objection of Parents and Medical Personnel

If all the above criteria for informed consent (or refusal) are met, parents can in some countries object to vaccination on personal, conscientious grounds (i.e., for religious, moral, or philosophical reasons (“non-medical exemptions”)) [3,41,51,59]. It is questionable if parents should be entitled to assert conscientious objection to vaccination, and, if so, what constraints this entitlement should be subject to [60,61]. Clarke et al. states that conscientious objection to vaccination for severe or highly contagious diseases is justified only in cases when the rate of its assertion does not threaten herd immunity [60]. In 2019 there were 45 states in the USA which allowed non-medical exemptions from immunisation [62]. Among European countries with mandatory vaccination policy, only two of them allowed non-medical exemptions in 2020 [63].

On the other hand, there also are physicians’ and other medical personnel’s autonomy [41,64,65]. It is exercised through right to refuse certain procedures or treatments on conscientious grounds. Because conscientious objection can contradict physician’s duties and may even limit patient’s access to health care, this right is subject to certain restrictions [64,66]. Some medical practitioners across the world refuse to treat children that are not vaccinated. Because this can affect access of unvaccinated children to health care, justification of applying conscientious objection in this case is debatable [67,68].

## 3. Nonmaleficence

The principle of nonmaleficence requires from a physician to not create harm to a patient or a research subject. It is one of the fundamental principles in medical ethics since Hippocrates and it is known by the maxim “Primum non nocere”; first, do no harm [41,69]. In accordance with this principle, it is first needed to evaluate the harm or risk for harm of the proposed intervention, which is acceptable only when outweighed by benefits [41,69]. Vaccination is a minimally invasive and safe procedure, but there are some important aspects that need to be assessed according to the principle of nonmaleficence.

### 3.1. Adverse Events

Though very safe, vaccines are not completely without risk [70]. Because they are usually administered to healthy children and are only potentially beneficial to them, any adverse event has a negative impact on the risk:benefit ratio [71,72]. The most of adverse events are mild (i.e., pain and swelling at the injection site, mild fever, rash or syncope) [71]. Anaphylactic reaction happens in approximately one per 1 million doses [70]. Some other rare severe adverse events are febrile seizures, immune thrombocytopenic purpura, disseminated varicella infection in varicella vaccine and intussusception in rotavirus vaccine [70,71,73]. Rarer adverse effects are mostly observed only after vaccine licensure, with continuous monitoring [71]. There are several independent bodies entitled for pursuance of this sensitive task; GACVS (Global Advisory Committee on Vaccine Safety) is based in Geneva and gives advice on vaccine safety to WHO, while two main programs in the U.S. are VAERS (Vaccine Adverse Event Reporting System), run by CDC and FDA, and Vaccine Safety Datalink (VSD) [71,74]. Because incorrect associations between certain vaccines and adverse effects were made in the past, a couple of myths have developed, which have fostered mistrust about vaccines [70]. A widely prevalent one is association between MMR vaccine and autism, which was rejected with strong evidence [70,75]. A solution to misconceptions about vaccines is providing reliable data on any possible adverse event and counselling parents about their concerns [71].

### 3.2. Contraindications for Immunisation

Although vaccines are appropriate for the vast majority of patients, there are particular groups which have a bigger risk to develop adverse events. By the principle of nonmaleficence, vaccination is contraindicated for them [71]. Contraindications can be temporary (i.e., current therapies (high dose steroids, chemotherapy …), pregnancy (for live attenuated vaccines) and reactivation of autoimmune disease) or permanent (i.e., allergy to vaccine’s ingredient, serious adverse events to a prior dose of vaccine and immunodeficiency (for live attenuated vaccines)) [71,76]. When there might be an increased risk for adverse effect, vaccination should be delayed or performed with caution in order to react properly [71]. Examples of precautions are a mild adverse event to a prior dose of vaccine and acute moderate to severe illness with or without fever [71,76]. Some conditions are wrongly perceived as contraindications (e.g., mild to moderate local reaction, current antimicrobial treatment, preterm birth and chronic diseases) [76]. Clear guidelines need to be accessible to everyone involved in vaccination in order to avoid such misconceptions and secure both safety and consistency in vaccination [7,76].

### 3.3. Vaccine Development

The principle of nonmaleficence needs to be applied also for a process of vaccine development and registration. Because this process is accompanied by uncertainties about possible adverse effects, a special attention needs to be paid in order to prevent harm to test subjects and future vaccine recipients [23,24,77,78]. Because children have limited capacity of understanding and may be subject to coercion, they are considered a vulnerable population [79]. Therefore, special attention and certain restrictions in trial design are needed in order to protect them [77,79]. On the other hand, children are owed optimal and novel treatment options, which makes clinical trials in the paediatric population unavoidable and essential [79,80].

## 4. Beneficence

The principle of beneficence defines a fundamental mission of healthcare providers—to contribute to the welfare of their patients [41,81]. It is a physician’s duty to apply a treatment or procedure that he or she recognizes as a patient’s best interest [81]. This principle is an upgrade of the principle of nonmaleficence, because it requires healthcare providers to take positive steps in helping their patients [41]. Beneficence is the basis for people’s trust in the healthcare system and medical personnel, which is necessary for a constructive patient–physician relationship and is crucial also for efficient immunisation programmes [82]. The goal of providing benefit can be applied not only to individual patients, but also to a society as a whole (e.g., in researches or vaccination) [41].

### From Individual’s to Public Health Benefit of Vaccination

Herd immunity has contributed to a dramatic decrease of infectious diseases or even to their eradication, and it has been clear that the benefit of immunisation strongly outweighs costs and risks [83]. But since in many countries’ vaccination programmes have been so successful, such that these diseases do not represent a direct risk anymore, the beneficial effect of vaccination has become less obvious [83]. When weighing risks and benefits of vaccination for an individual child in a population with low immunity rates or for non-communicable infectious diseases (e.g., tetanus and tick-borne meningoencephalitis), the benefits strongly outweigh risks [5]. On the other hand, when the level of herd immunity is high, vaccination of a single child has almost negligible beneficial effect for him or her [5]. However, because this is a result of an already established herd immunity, a risk:benefit analysis needs to include public health aspect (i.e., benefit for other individuals). Just contribution to benefits of vaccination is discussed in the following section.

## 5. Justice

The principle of justice is, in general, defined by two concepts: equitability and distributive justice [81]. Equitability means that persons in like circumstances are treated similarly, while distributive justice means fair distribution of limited healthcare resources among patients [81]. Criteria used in practice are not absolute but can change during time of emergency or crisis (e.g., in a pandemic, disease outbreaks), when public health perspective prevails over an individual-centred approach [84]. In public health, the principle of justice means ensuring subjects equal access to preventive measures and, additionally, equal contribution to control of communicable diseases (i.e., by vaccination) [85,86,87]. Giving up one’s own interests to serve the common good is the idea of solidarity, which cannot be considered as a synonym or alternative to the rights-based concept of justice, but as a necessary complement to it [88]. Features of solidarity are reciprocity and commitment to action, which brings also responsibilities to the participants [89]. Although vaccine allocation is an important issue, especially in the times of a pandemic, we will in this section focus on just contribution to herd immunity.

### 5.1. Need to Define Herd Immunity as a Common Good

Usual examples of common goods are drinking water, food safety, clean air, etc. Their features are that they are non-excludable and non-rival, which means that nobody can be excluded from their benefits and new subjects do not diminish their availability [5,72]. Since control of infectious diseases is a public interest, herd immunity should be recognised as a common good [2,11]. On the one hand, it means a form of solidarity with vulnerable members of our society; it helps to protect those, who are too young or too old to be vaccinated, unable to benefit from vaccination or have medical contraindications [11,90]. On the other hand, it allows so called “free riders” to enjoy benefits of herd immunity without bearing any costs of vaccination [90]. Distinctive for public goods is also their failure in ordinary marketing; therefore, they need to be maintained by external forces [72]. In our case, regulation of herd immunity could mean a mandatory vaccination policy. In addition, some similar common goods could even be recognised as constitutional rights (e.g., right to access to drinking water in Slovenia), which might even be a model for re-thinking herd immunity [91].

### 5.2. Ethical Justification of Obligatory Medical Procedure

Once herd immunity is established and the direct benefit of vaccination for an individual is low, the challenge is to maintain a sufficient level of immunity in a population [90]. Because education about risks and benefits is of limited value when herd immunity is achieved, it turned out in recent years that outbreaks of diseases (e.g., measles) can happen again in the countries with voluntary vaccination and not a sufficient degree of herd immunity [29,90,92]. To prevent outbreaks and protect also vulnerable members of society, obligatory vaccination is sometimes justified [5,61,87,93,94,95]. Gostin proposed conditions for ethical justification of obligatory medical procedure, which are as follows: Inefficient measure on a voluntary basis, absence of a less coercive alternative, unambiguous scientific grounds and presence of risk to the members of society, which they are unaware of [96]. He claims that restricting non-medical exemptions from vaccination is consistent with freedom of religion and conscience, because this policy does not target a particular community, but is applied equally throughout the society [61]. Similarly, Giubilini argues that individuals have a moral obligation to be vaccinated, although contribution of a single individual to the realisation of herd immunity is insignificant. When individuals do not fulfil their duty, coercive vaccination policies are justified. They should be implemented gradually by the principle of least restrictive alternative [5,78]. Flanigan also claims that compulsory vaccination is justified, because vaccine refusal can harm innocent people that are susceptible to certain diseases. He provides an analogy of vaccine refusal to randomly firing a gun [93]. On the other hand, some authors claim that solidarity should be a goal, not conformity. Parents should have a sense of moral responsibility, which is possible only if they are free to decide [29]. Coercive measures can have even an adverse effect, especially in the long term [7,82]. However, a French survey showed that implementation of mandatory vaccination in 2018 and simultaneous promotion strategies had a positive impact on the mothers’ opinions regarding vaccination [97]. To achieve adequate levels of responsibility, regardless of vaccination policy, a trustful dialogue between healthcare providers and parents should be established, as well as public deliberation to foster a sense of the community benefit of vaccination and to enable parents to participate as citizens [7,29,82].

### 5.3. State’s Responsibility and Policies

With very little or no dispute, the state is widely recognised to be entitled to regulate traffic safety bylaws to prevent the causalities. To better understand the state’s role in the regulation of herd immunity, we provide an ethical analogy of vaccination to traffic. Not using a child’s safety belt in the car or having a child cycling without a helmet endangers a child only. This resembles a situation when parents refuse a vaccine for non-communicable infectious disease (e.g., tetanus). Driving too fast endangers a child and passengers in other cars as well, which is analogous to parents refusing vaccine for a communicable disease (e.g., measles). In the case that most drivers drive slowly (because of their sense of responsibility or because of law), also the ones, who need to go faster (e.g., ambulance on an emergency service) are safer. This situation is comparable to a sufficient level of herd immunity. The state (through its formal structures and legislation) should be considered as a guardian of the public health [87]. This fact results in some similar implications for both maintaining traffic safety to reduce the injury rates (and thus fatalities) and herd immunity to reduce the rate of morbidity (and thus mortality) from infectious diseases; in both cases, behaviour of any individual potentially influences the health status of another individual within the population [98]. However, we acknowledge that this analogy is not ideal, because unlike measures for traffic safety (some of which might be unpleasant or restrict us physically), vaccination is an invasive procedure (though the risks are only minimal). Therefore, parents have the right and duty to evaluate possible burdens and ask for reassurance.

The best solution to achieve herd immunity is country-specific and reflects epidemiologic, cultural and healthcare system differences [19,61]. Though states are responsible to establish and maintain herd immunity, coercive measures should be kept to a minimal sufficient level [6,61,90]. Strategies to achieve herd immunity include positive measures that make vaccination more favourable (e.g., education or family tax benefit) and negative measures (e.g., financial fines or prohibition of entering a kindergarten or a school) [19,57,92]. The state’s responsibility for information disclosure and education about vaccination should be considered a crucial portion of its responsibility for maintaining herd immunity, regardless of implemented vaccination policy [30,56]. In 2014, the European Union Council adopted the conclusion on “vaccinations as an effective tool in public health”, which encouraged several countries to implement compulsory laws as a response to spreading anti-vaccination movements, which have threatened herd immunity in recent years [19,99]. A survey from 2020 evaluated mandatory vaccination policies in Europe, showing that they resulted in higher vaccination coverage and lower measles incidence [63]. In the United States, the school attendance is universally conditioned on compliance with a schedule of vaccinations [100]. However, compulsory policies should be considered as temporary rather than absolute, and constant reflection about their necessity and long-term effect is important [19,90]. A government should also take responsibility for a minority of individuals affected negatively by vaccination, especially of new, less tested vaccines [56]. When a causal link is proved, an economic or other compensation should be provided [56]. Responsibility of governing bodies towards control of infectious diseases should not be limited to a national level but extended to a global level. In providing measures (e.g., vaccination), the burden of the disease should take precedence over economic benefit. This requests development of vaccines for diseases, which do not necessarily represent significant burden in developed countries but are of major concern in developing parts of the world (e.g., Malaria, Tuberculosis, Ebola, AIDS) [101].

## 6. The Four Principles Practical Application: Two Clinical Vignettes

The four principles of biomedical ethics may be perceived by some of their critics as vague and their application as an oversimplification of often very complex real-life cases [102]. However, they have an important role as a tool to split complex ethical dilemmas into smaller, more comprehensive elements [102]. Better understanding of relevant aspects of a particular case might guide decisions and also help to achieve a reasonable degree of moral justification for an individual [40]. In this section, we provide two practical examples (case vignettes) on how the four principles can help both practitioner and patient to better reflect on the ethical dilemmas at stake, and to reach a sounder ethical decision in the context of vaccination in childhood.

### 6.1. Clinical Vignette #1

After an infant had received DTaP vaccine at the age of three and five months, the mother, a 24-year-old hairdresser, did not respond to invitations for the application of MMR vaccine at 12 months and delayed a systematic examination. The paediatrician called her and also asked directly whether she had some worries about vaccination, which she confirmed. The paediatrician invited her to come to the office to discuss her concerns. In the conversation, a physician found out that the child’s mother was not strictly against vaccination but was hesitant due to a couple of misinformation reasons. She has heard from her clients that vaccination is connected to autism, one acquaintance of hers even has a child that is disabled, supposedly because of vaccination.

Respect for autonomy: It is clear that the mother had been misinformed and lacks reliable information, which needs to be provided to her in order to meet criteria for parental permission (which are adequate information disclosure and understanding, decision making capacity and voluntariness). It is a physician’s duty to disclose information in such a way that she understands, including the information about possible risks and burdens of vaccination. It is crucial to build a trustful relationship, in which the mother feels comfortable to express her worries and can trust the paediatrician to work for her child’s benefit. Misinformation of the mother (connection of vaccines to autism and developmental disorders) could be effectively confronted in such relationship. Person-to-person consultation is shown to play a vital part in the vaccine uptake and should always be available to parents [103,104]. However, public health strategies with systematic provision of information about vaccination (i.e., on maternity wards; special leaflets) are an important complement to it, especially due to time limitations in the office [105].

Beneficence/nonmaleficence: The risk:benefit ratio assessment of vaccinating the infant with the MMR vaccine requires examination of possible contraindications or precautions (not existing in this case), but also overcoming any misconceptions about contraindications (autism was clearly shown not to be connected to vaccination, which needs to be reassured to the parents [75]). Public health benefit should also be implied, especially since measles require a high level of herd immunity, which also needs to be appropriately communicated to the family.

Justice: It is important that the family understands that the individuals with no contraindications have responsibility to be vaccinated (since the just contribution to herd immunity means just distribution of risks and burdens among population). Outbreaks of measles happened in recent years in many countries throughout the world (including nearby the region of the family residence); this scenario could be presented to the hesitant parents.

### 6.2. Clinical Vignette #2

A well-educated mother, aged 35, came to the office for a systematic examination of her three-month-old baby and clearly stated that she is not going to let him be vaccinated (the infant should receive the first dose of DTaP vaccine). She explained that she had adequately informed herself about vaccination and decided not to risk adverse events and pain of the needle, since there is no need to vaccinate her child due to absence of vaccine-preventable diseases. If she will need a vaccination certificate for a nursery, she will consider a private day care.

Respect for autonomy: The paediatrician firstly needs to ask the mother some questions to see how determined she is, and to make sure that her decision was autonomous, not based on misconceptions or a “controlling influence” by some other people. The paediatrician should know which strategies help in promotion of vaccination [4,106]; provision of simple facts about vaccination or paternalistic approach may backfire and make parents even more hesitant [107]. One efficient strategy is motivational interviewing, which aims to inform parents about vaccinations, according to their specific needs and their individual level of knowledge, with respectful acceptance of their beliefs [107].

Beneficence/nonmaleficence: The paediatrician needs to consider possible contraindications or precautions for vaccinating the child with DTaP vaccine. By the principle of beneficence, the paediatrician should be aware of the duty to protect the child (and by herd immunity also other children) against communicable diseases. She can explain to the mother that there is a significant risk for vaccine-diminished diseases to re-appear, as it has happened recently with measles. Whooping cough is actually present in population and is life-threatening for small infants. Though there is no herd immunity benefit of vaccination against tetanus, a risk of acquiring tetanus is bigger than the risk of vaccination, and; therefore, the benefit to the individual child is sufficient.

Justice: It requests just contribution to herd immunity and protection of vulnerable subjects; therefore, it counters the principle of respect for parents’ autonomy. Having the principle of justice in mind, the concept of vaccination as a partially altruistic medical procedure should be explained to the mother. She needs to be—with empathy—encouraged to consider the fair burden of vaccination also to protect others who might potentially be affected by her decision. This might be important for people that highly value the wellbeing of other people. In the case that the mother—after communicational interventions—still rejects vaccination, the ethical equilibrium between principles can be applied and a non-medical exemption can be made in case that the level of herd immunity is high enough and it is in accordance with the law (see Discussion). When there is not enough space to respect parents’ autonomy due to a high risk for the diseases to outbreak or due to high individual risk for a child, an external body, not a paediatrician, should be responsible to intervene in order to retain a trustful parents–paediatrician relationship.

## 7. Discussion

We applied the four principles of biomedical ethics framework to the ethics of vaccination in childhood to find the best possible balance among the principles to guide decision-making at the individual and at the societal level [41]. In the ethics of vaccination, there are two major conflicts: One is between respect for autonomy and best interest of an individual (autonomy vs. beneficence/nonmaleficence), the other is between respect for autonomy and public good (autonomy vs. justice) [6]. Because every dilemma by which physicians are confronted when parents refuse to vaccinate their children is context-specific and unique, we suggest a possible and simplified approach using the four principles.

Firstly, the principles of beneficence and nonmaleficence should be considered. What are the risks and benefits of vaccinating a particular child? Are there any contraindications or precautions? Serious adverse effects are extremely rare in the general population and benefits of vaccination strongly outweigh burdens [70]. When in doubt whether a certain condition is a possible contraindication, a risk:benefit assessment by an expert is indicated [76]. Summaries of vaccine product characteristics and guidelines are useful in decision-making [76].

The principle of autonomy requests to review criteria for informed consent/refusal (decision making capacity, adequate disclosure of information, understanding, voluntariness). Because most vaccine-hesitant parents lack reliable knowledge about vaccination or are misguided, open conversation about their dilemmas and possible risks can help them consent to vaccination [30]. It is also needed to recognize the “controlling influence” of other people’s opinions, conspiracy theories or anti-vaccination movements, which limits parents’ autonomy [2]. Many “dilemmas” can be solved through better communication and a systematic approach to vaccine education at all levels.

A principle of justice requests just contribution to herd immunity, which challenges respect for parental autonomy. Herd immunity should be recognised as a common good, possibly even as a constitutional right. It is of great importance to promote vaccination by “positive” strategies, which include responsible behaviour of medical personnel through example and endeavour, as well as promotion, education and provision of free vaccines at the national level [29]. Voluntary compliance with vaccination requirements is the most desirable, but when the moral duty of individuals to contribute to the common good is not fulfilled and herd immunity is endangered, “negative” vaccination policies (i.e., laws and penalties, vaccination mandates for nursery or school entrance etc.) are justified in order to protect vulnerable subjects [5,61].

A balance needs to be found between maintaining a sufficient level of herd immunity and respecting parents’ autonomy. We suggest finding an “ethical equilibrium”, which means that the degree of respect for parents’ autonomy is not constant, but variable; it shall depend on a level of established herd immunity [60,108]. In Figure 1, a curve which represents the relation between parents’ autonomy and benefit:risk ratio (or social justice) can be moved upwards in case of good society immunisation levels, which could allow non-medical exemptions. The principle of ethical equilibrium requests constant deliberation of risk for outbreaks and the need for mandatory vaccination policies.

## 8. Conclusions

Herd immunity may not be very obvious, but it is a precious common good, which is of benefit to everybody. However, once it is established, vaccination of a single individual is not very beneficial to the individual and risks may outweigh benefits. Therefore, vaccination should be considered as an altruistic procedure. When moral obligation of individuals to contribute to herd immunity is not fulfilled, mandatory vaccination policies are justified, because states bear the responsibility to protect herd immunity as a common good. Vaccination is, with its invasiveness into one’s body and possible (though minimal) risks, specific among mandatory measures and; therefore, respect for patients’ or parents’ autonomy is even more necessary. The optimal balance needs to be found between maintenance of herd immunity and parents’ autonomy; an ethical equilibrium, which is specific for every society. Regardless of vaccination policy, a constant reflection on level of herd immunity and mandatoriness of measures is needed. The priority goal of vaccination policies should be protecting herd immunity and, concurrently, building trust in vaccination and those who provide it. A summary of the framework for ethical analysis of vaccination in childhood is provided in Table 1.

## Figures and Tables

**Table 1:** A summary of a framework for ethical analysis of vaccination in childhood based on the four principles of biomedical ethics by Childress and Beauchamp [41].

RESPECT FOR AUTONOMY	NONMALEFICENCE
Respect for patient’s or parent’s will.	First, do no harm.
Criteria for parental permission: ○ Decision making capacity; ○ Adequate information disclosure; ○ Adequate understanding; ○ Voluntariness. Importance of trustful doctor–parents relationship, provision of reliable information, refutation of misconceptions, avoidance of “controlling influence” and proper risk communication; Conscientious objection (subject to restrictions!): ○ Of parents—to refuse vaccination on moral, religious or philosophical grounds (non-medical exemptions); Of physicians—to treat children whose parents refuse vaccination.	Adverse events of vaccination: rare and usually mild, severe ones are extremely rare; Contraindications: When risk is not outweighed by benefits; Precautions: When risk is slightly elevated (vaccination is delayed, or precaution is needed when vaccinating); Misconceptions: Conditions, that are wrongly perceived as contraindications; clear guidelines need to be accessible!
BENEFICENCE	JUSTICE
What is patient’s and society’s best interest?	Just distribution of benefits, risks and burdens.
Vaccination is one of the top public health achievements, saving millions of lives; Double beneficial effect of vaccines: ○ Direct protection in an individual; ○ Indirect protection through herd immunity. High level of herd immunity in a population diminishes beneficial effect of vaccination for a single individual, which makes vaccination an altruistic medical procedure.	Equal access to vaccination; Equal contribution to herd immunity as a common good: ○ Individuals have responsibility and duty to be vaccinated; ○ Herd immunity is non-excludable and non-rival; ○ Solidarity with vulnerable subjects; ○ opportunity to “free ride”. Conditions for ethical justification of obligatory vaccination policy: ○ Inefficient measure on voluntary basis; ○ Absence of less coercive alternative; ○ Risk of an infectious disease; ○ Unambiguous scientific grounds.
ETHICAL EQUILIBRIUM Between the principles, particularly respect for parents’ autonomy and just contribution to herd immunity; Specific for every society; Need for constant reflection on level of herd immunity and mandatoriness of coercive measures.	
